# Current News

**Published:** 1891-11

**Authors:** 


### Current News.

### AMERICAN ACADEMY OF DENTAL SCIENCE.

The Twenty-fourth Annual Meeting of the American Academy of Dental Science will be held in Boston on Wednesday, November 11, 1891, at 3 P.M.

The annual address will be delivered by George S. Allan, D.D.S., of New York.

E. N. Harris, D.D.S.,
*Corresponding Secretary.*

248 Boylston Street, Boston, Mass.

### ANNIVERSARY MEETING OF THE FIRST DISTRICT DENTAL SOCIETY.

The First District Dental Society of New York City will hold its usual anniversary meeting during the first week in January, 1892. The essayists are all eminent gentlemen, and the papers will be upon topics of vital interest. Specially-prepared discussions will be arranged, in which equally prominent men will participate.

It has been decided to manage the clinics upon a novel plan. Nothing will be offered that has ever been shown before. Gentlemen who can promise to exhibit some entirely new method, instrument, device, or medical agent are cordially invited to communicate immediately with Dr. F. A. Roy, 148 West Seventieth Street, chairman of Clinic Committee. Arrangements will be made so that every one shall have an opportunity to witness all demonstrations.

It is confidently predicted that we shall have with us, as usual, the majority of the prominent men of the country, and all are cordially invited to attend.

M. L. Rhein,
George H. Winkler,
Rodrigues Ottolengui, *Chairman*,
*Executive Committee.*

